# Readiness to change among involuntarily and voluntarily admitted patients with substance use disorders

**DOI:** 10.1186/s13011-019-0237-y

**Published:** 2019-11-06

**Authors:** Anne Opsal, Øistein Kristensen, Thomas Clausen

**Affiliations:** 1 University of Agder, Faculty of Health and Sport Sciences, Post-box 422, 4604 Kristiansand, Norway; 2 Sørlandet Hospital, Addiction Unit, Post-box 416, 4604 Kristiansand, Norway; 3 Norwegian Centre for Addiction Research (SERAF), Institute of Clinical medicine, University of Oslo, Post-box 1039, Blindern, 0315 Oslo, Norway

**Keywords:** Substance use disorder, Involuntary admission to treatment, Treatment motivation

## Abstract

**Background:**

Health care workers in the addiction field have long emphasised the importance of a patient’s motivation on the outcome of treatments for substance use disorders (SUDs). Many patients entering treatment are not yet ready to make the changes required for recovery and are often unprepared or sometimes unwilling to modify their behaviour. The present study compared stages of readiness to change and readiness to seek help among patients with SUDs involuntarily and voluntarily admitted to treatment to investigate whether changes in the stages of readiness at admission predict drug control outcomes at follow-up.

**Methods:**

This prospective study included 65 involuntarily and 137 voluntarily admitted patients treated in three addiction centres in Southern Norway. Patients were evaluated using the Europ-ASI, Readiness to Change Questionnaire (RTCQ), and Treatment Readiness Tool (TReaT).

**Results:**

The involuntarily admitted patients had significantly lower levels of motivation to change than the voluntarily admitted patients at the time of admission (39% vs. 59%). The majority of both involuntarily and voluntarily admitted patients were in the highest stage (preparation) for readiness to seek help at admission and continued to be in this stage at discharge. The stage of readiness to change at admission did not predict abstinence at follow-up. The only significant predictor of ongoing drug use at 6 months was SUD severity at baseline.

**Conclusions:**

The majority of involuntarily admitted patients scored high on motivation to seek help. Their motivation was stable at a fairly high level during their stay, and even improved in some patients. Thus, they were approaching the motivation stage similar to the voluntarily admitted patients at the end of hospitalization. Therapists should focus on both motivating patients in treatment and adapting the treatment according to SUD severity.

**Trial registration:**

ClinicalTrials.gov, NCT00970372. Registered 1 September 2008, https://clinicaltrials.gov/ct2/show/NCT00970372. The trial was registered before the first participant was enrolled. The fist participant was enrolled September 02, 2009.

## Background

Health care workers in the addiction field have long emphasised the importance of patient motivation to the outcome of treatment for substance use disorders (SUDs). Many patients entering treatment are not yet ready to make the changes required for recovery and are often unprepared or sometimes unwilling to modify their behaviour [1]. If lack of motivation is a common phenomenon in treatment, this may impact treatment outcomes [2, 3].

**Fig. 1:**
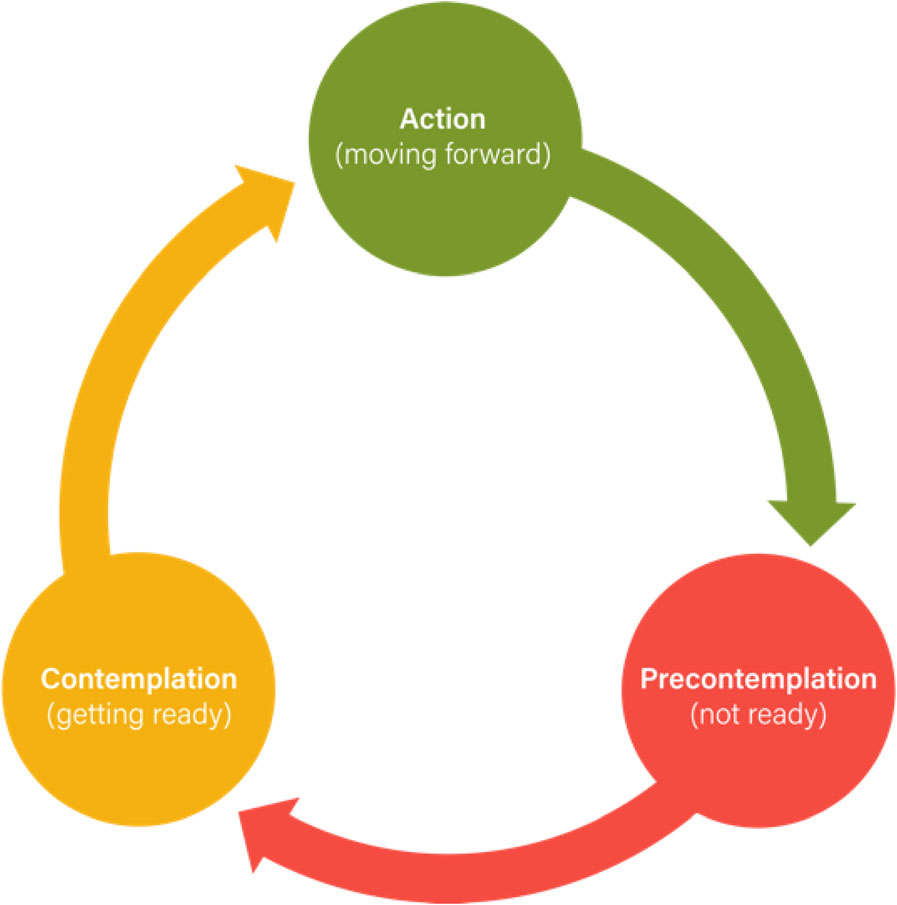
Modified Transtheoretical (Stages of Change) Model based on the theory that individuals follow a circular rather than linear path as they flow through a series of stages to modify behaviour (Modified from the work of Prochaska and DiClemente)

The most prevalent and widely used model to operationalise patient motivation to change in substance use treatment is the Transtheoretical Model [1]. The work of Prochaska and DiClemente on “stages of change” has become the conceptual and theoretical foundation for much of the work on motivation in the substance use field and in behavioural health in general. The model depicts categories in which patients tend to fall into when making significant changes in their lives, such as entering treatment. The model can be viewed as a continuum along which an individual may move toward long-lasting or permanent change. This model posits three stages that represent progressively greater commitment to change (Fig. 1) [4].

*Precontemplation* represents a stage in which there is little or no consideration of changing the current pattern of behaviour in the foreseeable future. Precontemplation is characterised by a lack of intent to change. The problem is not yet recognised as troublesome and/or may be seen as having more benefits than drawbacks for the person. *Contemplation* represents a stage in which the patients examine the current pattern of behaviour and potential for change in a risk-reward analysis. Contemplation is characterised by the individual thinking about the possibility of change, seeking and evaluating information, but not yet being fully prepared to change. *Action* represents the stage in which the patient implements the plan and takes active steps to change their current behaviour pattern and to begin creating a new behaviour pattern, such as entering treatment. Action is characterised by modification of the problem behaviour (e.g. protracted periods of abstinence, increased activity in therapy sessions, voluntary attendance of self-help meetings) and the development of new skill sets to prevent returning to the problem behaviour (e.g., help-seeking, use of Narcotics Anonymous or Alcoholics Anonymous sponsors, honestly talking about cravings).

To respond to harmful substance use, most countries provide some form of compulsory commitment to treatment [5, 6]. This is often motivated by the intent to protect an otherwise legally capable individual in a self-destructive and vulnerable situation due to substance use [5]. It is a controversial treatment option that should only be implemented after voluntary care has had unsuccessful results [5–8]. Norway also has a public health act that permits involuntary interventions for adult patients with SUDs. The act covers an option for retention of up to 3 months when voluntary efforts are insufficient, and the health of the patient is at serious risk because of extensive and prolonged substance use. In the acute phase of compulsory treatment, the main goal of retention is to provide life-saving treatment for the involuntarily admitted (IA) SUD patients. Over the longer term, the aim of treatment is to motivate patients to enter voluntary treatment and engage in change processes towards long-term recovery [9].

Legally coerced treatment remains controversial and poses a variety of challenges for health care workers [10]. This controversy often focuses on a debate about the effectiveness of coerced treatment [11, 12]. Concerns about the efficacy of legally coerced treatment for SUD stems from beliefs that coercion interferes with the ability to establish and maintain a therapeutic relationship that enables participants to benefit from treatment. Another concern is based on the notion that, to fully benefit from treatment, patients must be motivated to participate in treatment and change behaviour, and that the use of coercion disregards the importance of internal motivation in recovery. Compulsory commitment is the most consistent type of coercion, and involuntarily admitted patients may experience a type of “imprisonment”; there is a question of whether such an intervention is able to lead patients to more self-motivated actions towards recovery. Typically, it is considered to be crucially important that the treatment manages to change the patient’s motivation. Little research has examined the stages of motivation and compulsory commitment to treatment among patients with SUDs. The few studies have had mixed outcomes [2, 12–14], and the topic needs to be explored further.

The purpose of this study was to compare the stages of readiness to change (motivation) between SUD patients who entered treatment voluntarily and those who entered treatment involuntarily, and whether the stages of patient readiness to seek help changed during the treatment period. In addition, the patients´ experience of being involuntarily admitted to the hospital and factors associated with the ability to control drug use at 6 months of follow-up were investigated, with a focus on the patient’s readiness to change.

## Methods

### Study subjects

Consecutive patients were admitted to specialized units that offer treatment for patients with primary SUD often combined with mental disorders [15]. Patients recruited from one of three publicly funded treatment centres in the south-eastern part of Norway during the years 2009 to 2011 were eligible for inclusion in the study. The patients mainly came from urban and suburban areas. The IA patients were recruited from one of the centres in Kristiansand, Tønsberg, and Oslo that have four, four, and three beds for IA patients, respectively. All voluntarily admitted (VA) patients were recruited from the same ward of the Kristiansand centre.

The criteria for inclusion were: substance abuse or dependence in accordance with the International Classification of Diseases and Related Health Problems, 10th Revision (ICD-10) [16], age ≥ 18 years, understanding/speaking the Norwegian language, and at least 3 weeks of admission. Patients with mental retardation and/or not able to understand the questionnaires were excluded. Pregnant SUD patients were treated in separate wards and were not included in this study.

The study was approved by The Regional Committee for Research Ethics in Norway (REK 08/206d, 2008/2900, 09/2413) and by the Privacy Issues Unit, Norwegian Social Science Data Services (NSD no. 18782). Written informed consent was obtained from all study participants. The participants received no financial compensation except NOK 200 (approx. USD 23) to cover travel expenses at follow-up 6 months after discharge.

### Measurements

The three wards were organised similarly. All wards were multidisciplinary (psychiatrists, psychologists, social workers, occupational therapists, specialised nurses, and other trained staff) and had specialised units that offered treatment for patients with primary SUD, but often included mental disorders. All units treated both genders, but the majority of the patients were males. The patients could use communal areas, but the exterior doors were locked; however, most of the patients were allowed to leave the ward if accompanied by a staff member. Many patients received visits from friends and family. The patients had to give urine samples for drug-screenings both as routine procedure and by suspicion. Treatment included assessment of somatic and mental health, with diagnoses based on a structured interview and examination in accordance with the ICD-10, pharmacotherapy, cognitive milieu therapy, and individual motivation enhancement.

Before inclusion in the study, patients in both the IA and VA groups were detoxified, which was verified by negative urine tests for alcohol, opioids, central stimulants (amphetamines, methamphetamines, and cocaine), benzodiazepines, and cannabis, or when a minimum of 14 days were spent in detoxification to establish baseline values that were not influenced by withdrawal symptoms.

Sociodemographic variables were measured using the European Addiction Severity Index, a personal structured interview designed for both clinical and research purposes (Table 1). This index includes seven areas: medical status, employment and support status, drug and alcohol use, legal status, family history, family and social relationships, and psychiatric status [17]. In this study, we used the variables medical status, employment and support status, and drug and alcohol use. A Norwegian version of the European Addiction Severity Index-based interviews were performed by trained and certified staff. Injecting illicit drugs during the 6 months before admission and lifetime prevalence of overdoses were used as indicators of SUD severity.

**Table 1:** Instruments used in the study at admission, discharge and 6 months follow-up

	Admission	Discharge	6 months follow-up
ICD-10^a^	Clinical interview/observation		
European Addiction Severity Index	Clinical interview/observation		Clinical interview/observation
Readiness to Change Questionnaire	Questionnaire		
Treatment Readiness Tool	Questionnaire	Questionnaire	
Follow up interview			Clinical interview

^a^ICD-10: International Classification of Diseases and Related Health Problems, 10th Revision

Motivation is defined as the process that initiates, guides, and maintain goal-oriented behaviours, and is what causes one to act. We investigated the performance of two motivation measures that separately assess readiness to change (Readiness to Change Questionnaire; RTCQ) and readiness to seek help (Treatment Readiness Tool; TReaT). The RCTQ was originally developed for alcohol use and validated by Heather and Rollnick in 1993 [3]. It was modified by Burke and Gregoire to measure readiness to change for either alcohol or substance use [2]. In this study, an approved Norwegian forward-back translation of the Burke and Gregoire version was used. The validity of the questionnaire was verified by Rollnick et al. [4].

The patient’s readiness to change their stage of motivation was assessed at the onset of treatment. The RCTQ scale discriminates between three stages of motivation to change based on the Transtheoretical Model of behaviour. The RTCQ is a three-dimensional self-administered questionnaire assessing each of the stages with four item statements regarding the patient’s beliefs about their current alcohol and drug use [3]. The five-point scale ranges from strongly disagree (− 2) to strongly agree (+ 2). According to guidelines, if one of the four items on a scale is missing, the subject’s score for that scale [18] should be pro-rated (i.e., multiplied by 1.33). If two or more items are missing, the score cannot be calculated. In this case, the Stage of Change Designation will be invalid. Three patients were missing one of the four items and were prorated. One patient had two missing items and his answers were removed from the analysis. A negative score reflected overall disagreement with the items measuring the stage of change, whereas a positive score represented overall agreement. The highest score among the subscales represented the State of Change Designation. If two scale scores were equal, then the scale further along the continuum of change (precontemplation, contemplation, action) represented the subject’s Stage of Change Designation. We assigned patients to one stage based on their highest score.

Treatment readiness was measured at admission and discharge to treatment by the TReaT, which is based on the same theoretical model as the RCTQ. The TReaT may have advantages in predicting treatment compliance, and processes an outcome relative to the measure of general behaviour change readiness [19]. The TReaT is a 12-item self-report questionnaire referring to stages of formal help-seeking readiness: precontemplation, contemplation, and preparation. The item response scale is dichotomous (true/not true). A Norwegian translation was discussed and reviewed by recognised researchers in the assessment of SUDs. Translated items were then reviewed by the original authors of the assessment. Only complete questionnaires were included in the analysis.

Six months after discharge, we interviewed the IA patients. The patients were asked about their experience of being involuntarily admitted to the hospital. We used an interview guide with five questions: 1) Why were you involuntarily admitted to hospital? 2) Why did you not want treatment for your substance use prior to hospital admission? 3) What do you think afterwards about coercion being used? 4) What were your experiences from the actual involuntary admission? 5) What could have been done differently?

### Statistical analysis

Data were described by proportions (%), by median, quartiles (Q1, Q3) and range, or by mean and standard deviation (SD) when appropriate. The 95 percentage confidence interval (CI) for the difference in observed proportions between independent groups was calculated according to Wilson [20]. In the case of significant evidence of a difference, the test of differences in proportions corrected for continuity was performed [21]. The distribution of patient motivations on the three ordered levels according to the RTCQ assessed in the IA and VA patients were described by the median. The Wilcoxon-Mann-Whitney test of possible differences in motivation levels between the two independent groups was adjusted for tied observations [22]. The proportions of patients who changed their stages of motivation according to the TReaT and the difference in paired proportions with increased and decreased stages of motivation were calculated. The 95% CI for the difference in paired proportions estimates the expected change in motivation in a representative population [21].

A multiple linear regression model was used for modelling the dependence of the proportion of days abstinent the 30 days prior to follow-up on the variables SUD severity (injecting drug as proxy), motivation, and hospital admission type. The results are presented as β-values with 95% CIs [21]. Analyses were performed using SPSS 23.0 Software (SPSS Inc., Chicago, IL, USA).

The patients´ answers in the follow-up interview 6 months after discharge were analyzed using qualitative content analysis based on Granheim and Lundmann [23] and categorized into two categories. Numerical analyses were performed using Microsoft Excel.

## Results

A total of 103 consecutive IA patients were identified; 15 did not meet the inclusion criteria (12 because their stay was too short and 3 because of insufficient mental capacity) and 11 were not asked to participate because of logistical issues. Of the 77 patients eligible for inclusion, 12 refused to participate. Therefore, the rate of consent to participate among IA patients was 84% (65 patients). We identified a total of 223 VA patients; 72 were excluded (69 because their stay was too short and 3 because they lacked sufficient mental capacity). Of the remaining 151 VA patients, 14 refused to participate. Therefore, the rate of consent in the VA group was 91% (137 patients). The baseline characteristics of the 202 patients included in this study are given in Table 2. The different substances used were alcohol and illegal drugs like heroin, other opiates, benzodiazepines and other sedatives, amphetamines, cannabis, cocaine, inhalants, and hallucinogens. The mental statuses were divided in severe mental diagnoses (F20-F39), other mental diagnoses (F40-F99) and no mental diagnosis. A larger proportion of patients in the IA group than the VA group injected illicit drugs 6 months prior to admission to the study (*p* = 0.01). There was also a difference in proportion of patients that experienced overdoses during their lifetime (*p* = 0.02). In addition, a larger proportion of IA patients than VA patients were treated by a physician for somatic complaints 6 months prior to admission. However, a greater proportion of VA patients than IA patients attempted suicide at some point in their lives (proxy severe mental health symptom burden).

**Table 2:** Baseline sociodemographic variables and mental stress scores for involuntarily admitted or voluntarily admitted patients

	Involuntary n/Voluntary n	Involuntary	Voluntary
Age, years			
Median	65/137	24	28
Q_1_, Q_3_	65/137	21.0, 34.5	23.5, 36.0
Range	65/137	18.0–57.0	19.0–61.0
Female, n (%)	65/137	31 (48)	37 (27)
Substance use^b, d^			
Alcohol	60/132	29 (48.3)	41 (31.1)
Heroin	61/134	21 (34.4)	18 (13.4)
Other opiates	60/130	11 (18.3)	25 (19.2)
Benzodiazepines, other sedatives	60/134	39 (65.0)	63 (47.0)
Amphetamines	62/135	35 (56.5)	67 (49.6)
Cannabis	61/133	32 (52.5)	71 (53.4)
Cocaine, inhalants, hallucinogens	60/132	12 (20.0)	18 (13.6)
Mental diagnosis			
No mental diagnosis	65/137	26 (40.0)	35 (25.5)
Severe mental diagnoses (F20-F39)	65/137	14 (21.5)	38 (27.7)
Other mental diagnoses (F40-F99)	65/137	25 (38.5)	64 (46.7)
Education			
Mean years in primary school and high school (SD)	59/130	10.5 (1.4)	10.6 (1.6)
Patients attending college and university, *n* (%)	59/130	4 (7)	14 (11)
Sources of financial support^a, b^, *n* (%)			
Employment	60/130	6 (10)	24 (19)
Public welfare benefits	62/135	59 (95)	115 (85)
Partner, family, or friends	60/130	17 (28)	37 (29)
Illegal activity	60/130	24 (40)	47 (36)
Living arrangement^b^, *n* (%)			
With partner	59/130	8 (13)	11 (9)
Alone	59/130	31 (52)	62 (48)
With family	59/130	9 (15)	26 (20)
No stable arrangements	59/130	9 (15)	16 (12)
Controlled environment	59/130	2 (3)	15 (12)
Treated by a physician for somatic diseases^b^, *n* (%)	60/130	24 (40)	32 (25)
Injecting illicit drug^b^, *n* (%)	61/134	43 (71)	62 (46)
Overdoses on drugs^c^, *n* (%)	59/130	41 (70)	63 (49)
Suicide attempts^c^, *n* (%)	60/131	23 (38)	71 (54)

Q1, Q3: first, third quartile, SD: standard deviation. ^a^Some patients had more than one source of financial support,

^b^Last 6 months before admission, ^c^Lifetime prevalence, ^d^Some patients had more than one substance use

### Readiness to change

**Fig. 2:**
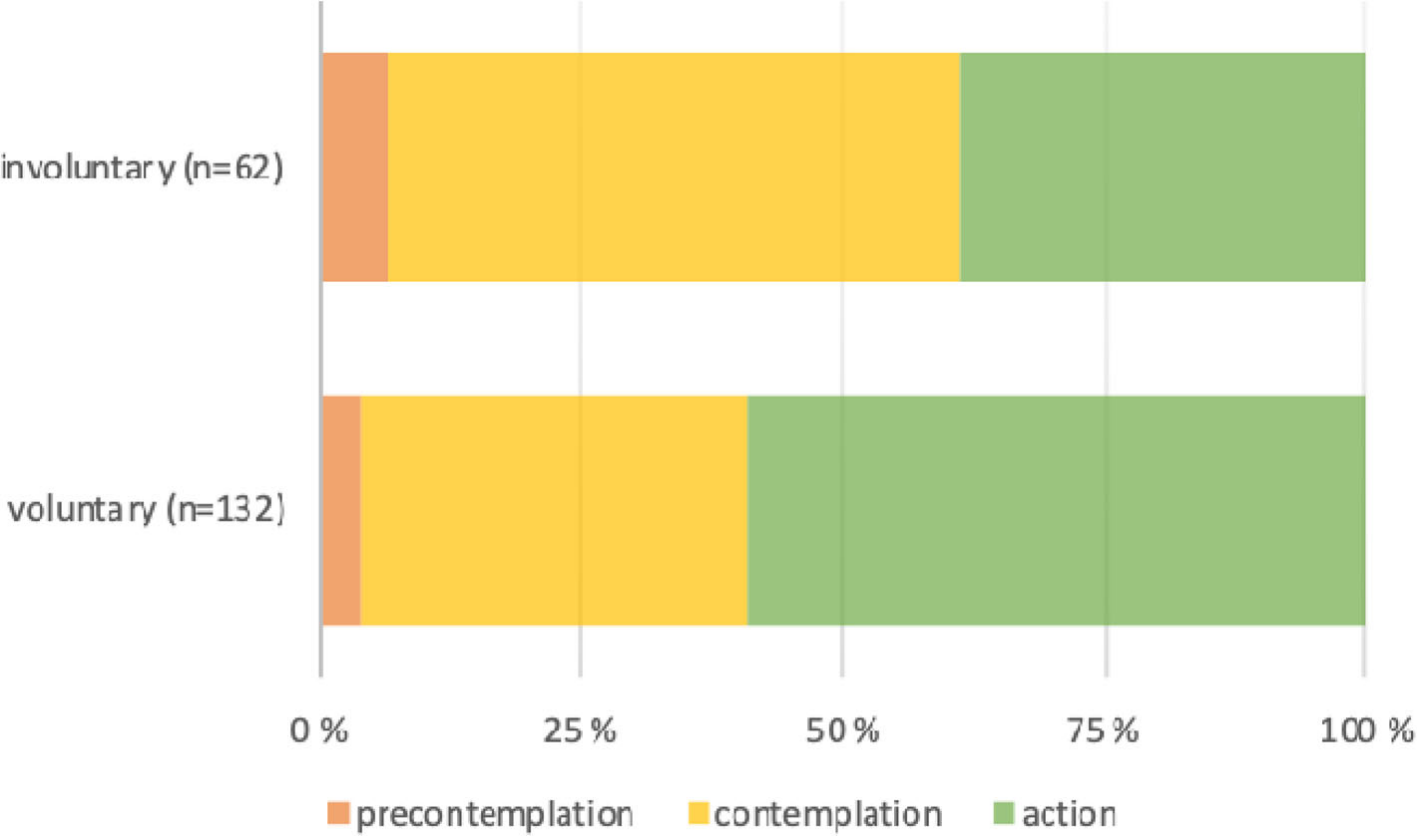
Percentage of patients admitted to the hospital based on stage of Readiness to Change

At admission, 39% of the IA group was in the stage of action vs. 59% of the VA group (Fig. 2). Significantly more VA patients than IA patients had a high score on action (*P* = 0.025). The IA patients scored significantly lower levels of motivation than the VA patients, with the median levels being contemplation and action, respectively (*p* = 0.008).

### Change in stage of readiness to seek help

**Fig. 3:**
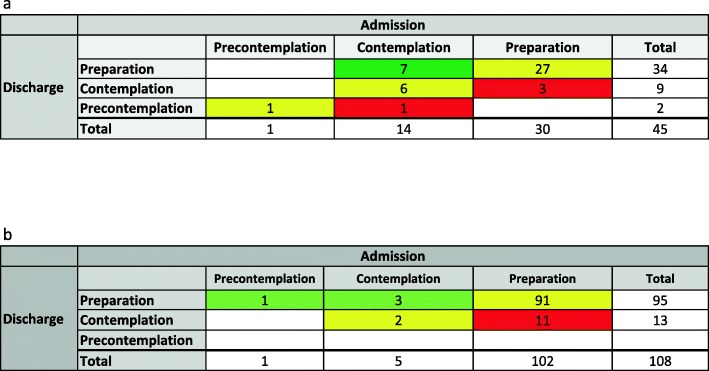
Changes in Treatment Readiness Tool (TReaT) stage from admission to discharge from addiction treatment centres. **a** Involuntarily admitted patients (*n* = 45). **b** Voluntarily admitted patients (*n* = 108). Unchanged stage: Yellow, changed to lower stage: Red, changed to higher stage: Green

The majority of involuntarily admitted patients scored high on motivation to seek help. Their motivation was stable at a fairly high level during their stay, and even improved in some patients. Four of the 45 IA patients changed to a lower stage and 7 to higher stages of readiness to seek help during treatment (Fig. 3a). The majority remained in the highest stage (i.e., preparation) throughout the involuntary treatment period. This means an estimate of 6.7 percentage units for increased readiness to seek help among these patients (non-significant). Corresponding results for the VA patients were 11 of the 108 patients changing to lower and 4 to higher stages of readiness to seek help. Thus, the involuntarily admitted patients were approaching the motivation stage similar to the voluntarily admitted patients at the end of hospitalization. None of the above findings represent significant changes in readiness to seek help.

In multiple linear regression analysis, only injecting drug use reported at admission was a significant explanatory variable for ongoing drug use at follow-up. Age, gender, readiness for change, or admission formalities were not significant explanatory variables (Table 3).

**Table 3:** Multiple regression analysis of the proportion of abstinence days 30 days prior to follow-up

Characteristic	Coefficient b	*P*-value	(95% CI)
Constant	72.5	0.001	(40.3 to 104.8)
Injected drug abuse^a^	−25.5	0.002	(−41.7 to −9.2)
Involuntary hospital admission	−16.0	0.076	(−33.7 to 1.7)
Readiness for change (RCTQ)	14.0	0.083	(−1.9 to 29.9)
Age	0.2	0.696	(−0.7 to 1.0)
Gender	−8.6	0.318	(−25.5 to 8.4)

^a^During the 6 months prior to admission

At follow-up 6 months after discharge, we performed new interviews with the IA patients to ask about their experience of being involuntarily admitted to the hospital. Of the 48 patients, 36 (75%) said that they acknowledged, in retrospect, that they needed help, and that they were happy that someone cared. Twelve (25%) of the patients were negative to the involuntary admission and had negative experiences overall. Most of these 12 patients experienced their admission as humiliating, especially when/if uniformed police were involved.

## Discussion

The IA patients scored significantly lower levels of motivation to change on the RTCQ than the VA patients at admission. However, many patients in both groups were in the highest stage (preparation) of readiness to seek help (TReaT) at admission and continued to be in this stage at discharge. The only significant predictor of ongoing drug use at 6 months was SUD severity at baseline. Stage of readiness to change at admission did not predict abstinence at follow-up.

According to the Norwegian Public Health Act (§10.2), legal coercion into treatment for adult patients with SUDs can only occur when voluntary efforts are insufficient, the patient rejects treatment, and the health of the patient is at serious risk due to extensive, prolonged substance use [9]. Looking at the law’s criteria for involuntary admission, IA patients are expected to be less motivated for change. The highest stage (action stage) represents the stage in which the patient implements the plan and takes active steps to change their current behaviour pattern and to begin creating a new behaviour pattern. Although a significant difference was found between the IA and VA groups, it was unexpected that so many IA patients were in this action stage. The precontemplation stage represents a stage in which there is little or no consideration to change the current pattern of behaviour in the foreseeable future. Precontemplation is characterised by the lack of intent to change. Considering that the IA patients had previously refused treatment, it is assumed that several of the IA patients would be in the precontemplation stage, but relatively few IA and VA patients were in fact in this stage. This could be explained by ambivalence, resistance to treatment because of poor experience with prior treatment, or psychological defence mechanisms, such as denial, trifling, and projection [24].

The longer term aim of involuntary treatment is to motivate the patients to subsequently enter voluntary treatment programs and engage in change processes towards long-term recovery [9]. Other studies have shown that readiness to change and readiness to seek help are not congruent [19, 25, 26]. At the time of admission, two-thirds of the IA patients reported that they were now motivated for a treatment they had rejected earlier. An explanation may be ambivalence, as the patient may have acknowledged that drugs are destroying his/her life, but he/she could not leave behind the drug-taking behaviour. Ambivalence is often prominent in SUDs [27]. In the IA group, the readiness to seek help increased during treatment, corresponding to the IA patients’ answers at follow-up 6 months after discharge when asked about their experience of being involuntarily admitted to the hospital. Thus, motivation is not static and may vary with time. Patients can change from one moment to another according to perceived environmental stimuli and also from internal motivation. The Stages of Change Model does not take into account the environment surrounding the individual [28]. Outside forces can easily affect the ability of people to change either by supporting continued use or by creating barriers to positive behavioral change. SUD is perceived as a chronic progressive disorder requiring treatment over time. The purpose of the Norwegian Public Health Act is for IA patients to be detained for up to 3 months while trying to improve their motivation for further voluntary treatment. This study shows stability and a slight increase in motivation for treatment during a stay, but there is still potential for further work with motivational issues during treatment. Clinical experience shows that it takes time to change drug use behaviours. Swedish legislation [29] permits withholding for up to 6 months. Thus, Swedish institutions have a greater opportunity to facilitate improvements for motivation change with legal coercion into treatment.

We were interested in investigating whether stages on the RTCQ before admission could predict the outcome of drug control (proportion of abstinent days last 30 days) prior to follow-up 6 months post-discharge. Based on previously published results by Heather et al. [3], we expected that a high stage of motivation to change (stage of action) would predict a better outcome. Our expectation failed. In the linear multiple regression analysis, we found that severity of SUD (injecting drug use as reported at admission) was the only significant explanatory variable for ongoing drug use at follow-up.

Burke and Gregoire found that IA patients are more likely than VA patients to report abstaining from alcohol and other drugs in the 30 days before their follow-up interviews. They were also more likely to demonstrate reduced addiction severity at follow-up. Readiness to change at admission exhibited no relationship with treatment outcomes [14]. IA patients reported better outcomes at the 6-month follow-up, even when taking into account the differences in RTCQ stage and addiction severity at admission [14]. Myers et al. found that the Stages of Change, Readiness, and Treatment Eagerness Scale (SOCRATES) predicted reduced substance use in a randomised controlled trial of patients attending South African emergency departments [30]. The only variable associated with change in substance use involvement from baseline to follow-up was baseline SOCRATES score. Heather and colleagues found that the stage of change was an accurate predictor of alcohol consumption among heavy drinkers at the 6-month follow-up [3]. For a lasting change, new behaviours and new habits must be incorporated, and that takes time (usually months). Therefore, motivation itself is not enough to make a change.

The outcomes of studies on motivation as a predictor of change in drug use and drug control are mixed. Most of the studies have been performed with VA patients. However, our study, like Burke et al. [14], examined both VA and IA patients.

### Limitations

These findings should be considered in light of some limitations. First, the sample size was relatively small, limiting the power to identify group differences. The sampling was limited to participants recruited from three addiction centres in southern Norway. However, we included a high number of IA patients relative to the total annual number of IA patients nationally. This increases the confidence in our findings, which may be representative of the situation in other areas in Norway. Second, the follow-up period was relatively short and whether the effects of readiness to change on substance use involvement could vary over time is unknown. Therefore, we could not exclude the possibility that the high percentage of individuals in the action stage found in the involuntarily admitted patient group could be explain by the fact that the RTCQ did not accurately measure the stages of readiness to change. This could also be a possible explanation for why the stage of readiness to change at admission did not predict abstinence at follow-up.

### Clinical implications

Even if our study could not confirm that stage of readiness to change prior to admission predicts drug control 6 months after discharge, using some of the motivation scales will be beneficial in clinical work, regardless of whether the therapy is Motivation Interview, Motivation Enhancement, or others. Clinicians may use motivation assessment for both clinical purposes and the prediction of those who need extra monitoring due to increased risk of premature attrition.

Furthermore, our study indicates that the IA patients need treatment adapted primarily to the severity of the disease. This means that the treatment should, to some extent, disregard the stage of motivation upon admission. Good mapping and investigation provide the basis for work with motivation, change, and prevention of dropout during treatment. Therapeutic conversations, activities, and meetings that contribute to positive experiences can be important.

The majority of IA patients in our study were admitted to the same department as VA patients, who had higher average scores on the motivation scales. Joint companionship is thought to positively influence the effect between the two groups and may explain the stage of motivation for treatment of IA patients approaching that of the VA patients during their stay. Active user involvement at the individual level also improves the ability of a good therapist/patient relationship and helps achieve the goal of the law regarding facilitation for further voluntary treatment. Well-planned admissions, where the patient is familiar with the decision about compulsory treatment and has participated in the process, is thought to improve the possibilities for motivating further treatment.

If the IA patient wishes further voluntary treatment, this should be offered promptly. Information on the place and selection of treatment, as well as repeated attempts to involve the patient despite resistance, improves the results by coercion [31]. Use of coercion also seems to prevent disability and contribute to functional improvement in quality of life for a longer period of time [32].

## Conclusions

We found a difference between the IA and VA patients with both the RTCQ and TReaT. An unexpected finding was that the majority of IA patients were motivated to seek help at treatment admission, and that their motivation was stable at a fairly high level during their stay, and even improving for some of the patients. Thus, they were approaching the motivation stage of the VA patients. Further studies with a larger sample size and longer follow-up should be performed to confirm the results of this study.

In the future, it will be important for therapists to focus on motivating patients for further treatment and adapting the treatment to their SUD severity.

## Data Availability

The data used in this study forms the basis of a still ongoing study that will be finalised in 2019. According to current Norwegian regulations and practice, the data will be anonymised December 31, 2019, and will then be deposited in the publicly available data repository of the Norwegian Centre for Research Data.

## Abbreviations

IA: Involuntarily admitted
ICD-10: International classification of diseases and related health problems, 10th revision
RTCQ: Readiness to change questionnaire and treatment
SUD: Substance use disorder
TReaT: Readiness tool
VA: Voluntarily admitted

## References

[1] Prochaska JO, DiClemente CC, Norcross JC (1992). In search of how people change: applications to addictive behaviors. Am Psychol.

[2] Gregoire TK, Burke AC (2004). The relationship of legal coercion to readiness to change among adults with alcohol and other drug problems. J Subst Abus Treat.

[3] Heather N, Rollnick S, Bell A (1993). Predictive validity of the readiness to change questionnaire. Addiction.

[4] Rollnick S, Heather N, Gold R, Hall W (1992). Development of a short 'readiness to change' questionnaire for use in brief, opportunistic interventions among excessive drinkers. Br J Addict.

[5] Israelsson M, Gerdner A (2010). Compulsory comittment to care of substance misusers: a worldwide comparative analysis of the legislation. Open Addiction J.

[6] Israelsson M (2011). Welfare, temperance, and compulsory commitment to care for persons with substance misuse problems: a comparative study of 38 European countries. Eur Addict Res.

[7] Urbanoski KA (2010). Coerced addiction treatment: client perspectives and the implications of their neglect. Harm Reduct J.

[8] Wild TC, Newton-Taylor B, Alletto R (1998). Perceived coercion among clients entering substance abuse treatment: structural and psychological determinants. Addict Behav.

[9] LOV 2011-06-24 nr 30: Lov om kommunale helse- og omsorgstjenester m.m. [Helse- og omsorgstjenesteloven], [The Act of public health and care services] [http://www.lovdata.no/all/hl-20110624-030.html].

[10] Nilssen E (2005). Coercion and justice: a critical analysis of compulsory intervention towards adult substance abusers in Scandinavian social law. Int J Soc Welf.

[11] Tvångsvård vid missbruk - effekt och kvalitet (Review on compulsory care for substance misuse – Effect and quality) [http://www.sou.gov.se/missbruk/pdf/Rapporter/Oversikt_tvangsvard_missbruk.pdf].

[12] Klag S, O'Callaghan F, Creed P (2005). The use of legal coercion in the treatment of substance abusers: an overview and critical analysis of thirty years of research. Subst Use Misuse.

[13] Pasareanu AR, Vederhus JK, Opsal A, Kristensen O, Clausen T (2016). Improved drug-use patterns at 6 months post-discharge from inpatient substance use disorder treatment: results from compulsorily and voluntarily admitted patients. BMC Health Serv Res.

[14] Burke AC, Gregoire TK (2007). Substance abuse treatment outcomes for coerced and noncoerced clients. Health Soc Work.

[15] Opsal A, Kristensen O, Larsen TK, Syversen G, Rudshaug EB, Gerdner A, Clausen T (2013). Factors associated with involuntary admissions among patients with substance use disorders and comorbidity: a cross-sectional study. BMC Health Serv Res.

[16] Janca A, Ustun TB, Early TS, Sartorius N (1993). The ICD-10 symptom checklist: a companion to the ICD-10 classification of mental and behavioural disorders. Soc Psychiatry Psychiatr Epidemiol.

[17] Kokkevi A, Hartgers C (1994). European addiction severity index EuropASI.

[18] Heather N, Rollnick S: Readiness to change questionnaire: user’s manual (revised version). In: *Technical Report No 19.* 1993.

[19] Freyer J, Tonigan JS, Keller S, John U, Rumpf HJ, Hapke U (2004). Readiness to change versus readiness to seek help for alcohol problems: the development of the treatment readiness tool (TReaT). J Stud Alcohol.

[20] Newcombe R, Altman D: Proportions and their differences. In: *Statistics with Confidence: Confidence Intervals and Statistical Guidelines, 2nd Edition.* edn. Edited by Altman D, Machin D, Bryant T, Gardner M: BMJ Books; 2000: 45–56.

[21] Altman DG (1991). Practical statistics for medical research.

[22] Siegel S, Castellan NJ: Nonparametric statistics for the behavioral sciences. 2nd ed. New York: Mcgraw-Hill Book Company; 1988.

[23] Graneheim UH, Lundman B (2004). Qualitative content analysis in nursing research: concepts, procedures and measures to achieve trustworthiness. Nurse Educ Today.

[24] Shaffer HJ, Simoneau G (2001). Reducing resistance and denial by exercising ambivalence during the treatment of addiction. J Subst Abus Treat.

[25] Freyer-Adam J, Coder B, Ottersbach C, Tonigan JS, Rumpf HJ, John U, Hapke U (2009). The performance of two motivation measures and outcome after alcohol detoxification. Alcohol Alcohol.

[26] Freyer J, Tonigan JS, Keller S, Rumpf HJ, John U, Hapke U (2005). Readiness for change and readiness for help-seeking: a composite assessment of client motivation. Alcohol Alcohol.

[27] Johnson V (1986). Intervention: how to help someone who doesn't want help.

[28] Michie S, van Stralen MM, West R (2011). The behaviour change wheel: a new method for characterising and designing behaviour change interventions. Implement Sci.

[29] SOU: LVM-utredningens betänkande (2004). "Tvång och förändring". In: *Socialdepartementet.* Edited by change.

[30] Meyers B, van der Westhuizen C, Naledi T, Stein D, Sorsdahl K. Readiness to change is a predictor of reduced substance use involvement: findings from a randomized controlled trial of patients attending south African emergency departments. BMC Psychiatry. 2016. 10.1186/s12888-016-0742-8 PMC4761196 26897614

[31] Lundeberg I, Mjåland K, Søvig K (2014). Tvang i rusfeltet - Regelverk, praksis og. erfaringer med tvang.

[32] Pasareanu AR, Opsal A, Vederhus JK, Kristensen O, Clausen T (2015). Quality of life improved following in-patient substance use disorder treatment. Health Qual Life Outcomes.

